# Cross-Cultural Adaptation and Psychometric Evaluation of the Perceived Ability to Cope With Trauma Scale in Portuguese Patients With Breast Cancer

**DOI:** 10.3389/fpsyg.2022.800285

**Published:** 2022-02-16

**Authors:** Raquel Lemos, Beatriz Costa, Diana Frasquilho, Sílvia Almeida, Berta Sousa, Albino J. Oliveira-Maia

**Affiliations:** ^1^Champalimaud Research and Clinical Centre, Champalimaud Foundation, Lisbon, Portugal; ^2^ISPA — Instituto Universitário de Ciências Psicológicas, Sociais e da Vida, Lisbon, Portugal; ^3^NOVA Medical School, NMS, Universidade Nova de Lisboa, Lisbon, Portugal; ^4^Breast Unit, Champalimaud Clinical Centre, Champalimaud Foundation, Lisbon, Portugal; ^5^Graduate Programme in Clinical Psychology, Faculdade de Psicologia da Universidade de Lisboa, Lisbon, Portugal; ^6^Ph.D Programme in Health Data Science, Faculdade de Medicina da Universidade do Porto, Porto, Portugal

**Keywords:** cross-cultural adaptation, validity, psychometrics, cancer, trauma, coping flexibility

## Abstract

**Background:**

The impact of a cancer diagnosis may be traumatic, depending on the psychological resources used by patients. Appropriate coping strategies are related to better adaptation to the disease, with coping flexibility, corresponding to the ability to replace ineffective coping strategies, demonstrated to be highly related with self-efficacy to handle trauma. The Perceived Ability to Cope with Trauma (PACT) scale is a self-rated questionnaire that assesses the perceived ability to cope with potentially traumatic events, providing a measure of coping flexibility. The current study aimed at examining the psychometric properties of the PACT Scale in Portuguese patients with breast cancer.

**Methods:**

The study included 172 patients recently diagnosed with early breast cancer. Participants completed a Portuguese version of the PACT scale, and instruments of self-efficacy for coping with cancer (Cancer Behavior Inventory-Brief Version—CBI-B), of quality of life (European Organization for Research and Treatment of Cancer Quality of Life Questionnaire Core-30—QLQ-C30), and of psychological distress (Hospital Anxiety and Depression Scale—HADS) that were used as convergent and divergent measures, thus assessing construct validity. A confirmatory factor analysis (CFA) was performed to test the factor structure of the Portuguese version of PACT scale and reliabilities were examined.

**Results:**

Results from the CFA confirmed the two-factor structure, consistent with the original Forward and Trauma focus subscales. The two subscales demonstrated high internal consistencies. Convergent and divergent validities were confirmed: the PACT scale was related to high self-efficacy to cope with cancer (CBI-B), to high perceived quality of life (QLQ-C30), and to low psychological distress (HADS).

**Discussion:**

Overall, the current results support and replicate the psychometric properties of the PACT scale. The scale was found to be a valid and reliable self-reported measure to assess Portuguese breast cancer patients regarding beliefs about their capabilities in managing the potentially traumatic sequelae of cancer. The PACT is a simple and brief measure of coping flexibility to trauma, with potential relevance for application in clinical and research settings.

## Introduction

The burden of cancer incidence in 2020 is estimated to have risen to 19.3 million new cases worldwide (Sung et al., 2021). Female breast cancer was the leading form of cancer globally in 2020, with an estimated number of 2.3 million new cases worldwide, representing 11.7% of all cancer cases (Sung et al., 2021). Among women, breast cancer accounts for 1 in 4 cancer cases, ranking first for incidence in the majority of countries (Sung et al., 2021). According to the World Health Organization Global Cancer Observatory, in Europe breast cancer was responsible for 12.1% of all new cancer cases in 2020, representing 25.8% of all female cancers.^1^ In Portugal, for example, the number of female breast cancer cases in 2020 was 7,041, representing 26.4% of cancer diagnoses in women (see text footnote 1).

Being diagnosed with cancer and experiencing cancer treatment is highly stressful, with the potential to become a traumatic experience, threatening physical and psychological wellbeing. Emotional reaction to this experience includes acute responses of fear, sadness, and anger, but also long-term adjustment difficulties characterized by anxiety and depression (Foster et al., 2009). Several stressors are associated with cancer diagnosis and the treatment trajectory. Uncertainty about prognosis, management of clinical information and decision-making regarding treatments can make the early phases of the cancer path particularly overwhelming (Hack et al., 2010). In the specific case of female breast cancer, the diagnosis may additionally challenge identity, self-esteem, body image and relationships (Campbell-Enns and Woodgate, 2015). Women who experienced distress due to breast cancer are at higher risk of feeling an impact on long-term quality of life, with estimations of 20–30% of survivors reporting psychological difficulties that persist for years after the diagnosis (Foster et al., 2009). Nevertheless, while a considerable proportion of people may experience cancer diagnosis and treatments as traumatic, this is not true for everyone (for meta-analytic reviews see Abbey et al., 2015; Swartzman et al., 2017).

These findings led to research on the prevalence, predictor factors and correlates of cancer-related post-traumatic stress disorder (PTSD) symptoms (Foster et al., 2009). Facing a cancer diagnosis differs from typical acute traumatic events: the stressor comes from an internal, rather than an external, locus, and individuals deal constantly with the presence of an ongoing threat, as opposed to experiencing a single past-incident traumatic event (Pat-Horenczyk et al., 2016). Studies exploring cancer as a traumatic stressor have used the DSM-IV-TR (American Psychiatric Association, 2000) PTSD diagnostic criteria to understand if patients experienced cancer diagnosis and treatments as a threat to their life or physical integrity (criterion A1) and if they reacted with fear, helplessness or horror (criterion A2). Across studies, 50–60% of patients endorsed the two criteria, with the first criterion endorsed more commonly than the second (Cordova et al., 2001, 2007, 2017). Among patients with cancer, Matthews et al. (2017) found that PTSD symptoms were more frequently reported by women than men (27% vs. 10%). The predictors of PTSD among women included perceived intensity of cancer treatment, difficulties with health care professionals, and using cognitive avoidant coping styles. For men the only predictor was behavioral avoidance (Matthews et al., 2017). Mehnert and Koch (2007) reported that cancer was a traumatic stressor for 54% patients with breast cancer, with patients scoring high in avoidant symptoms just after receiving the diagnosis presenting difficulties in adjustment up to 2 years later (Arnaboldi et al., 2017).

Since the impact of a cancer diagnosis varies according to the psychological resources that patients use, understanding how patients cope and adjust to a cancer diagnosis is essential to plan care. Macía et al. (2020) showed that, in people with cancer, the use of appropriate coping strategies and the presence of higher levels of resilience were related to better quality of life and better adaptation to the disease. The ability to engage in adaptive coping behaviors predicts optimal adjustment in the presence of highly aversive or potentially traumatic life events (Bonanno, 2004, 2005), such as after receiving a cancer diagnosis. Moreover, while active or instrumental coping strategies, such as positive thinking or dealing actively with problems, are associated with a positive adaptation to stress, passive coping strategies (i. e., avoidance) are usually considered maladaptive (for a review see Linley and Joseph, 2004; Cheng et al., 2014). Understanding the relationship of resilience and coping with quality of life represents valuable information for psychologists working in the oncological setting. At a practical level, it may implicate working with patients in modifying the type of coping, as well as increasing the level of resilience, toward achieving better adjustment to cancer.

In the specific case of breast cancer, self-efficacy to cope with cancer tends to improve over time after diagnosis (Kochaki Nejad et al., 2015), and has been associated to many well-being outcomes through a combination of cognitive, emotional, and behavioral variables (Karademas et al., 2021). Furthermore, coping self-efficacy has been shown to mediate the relationship between illness perception and fear of progression (Shim et al., 2018) as well as between perceived social constraints and symptoms among long-term survivors (Adams et al., 2017). Kant et al. (2018) observed that coping self-efficacy following breast cancer diagnosis predicts less psychological distress over time and a recent review confirmed that self-efficacy can predict quality of life and psychological distress in patients with breast cancer, thus highlighting that, at the time of diagnosis, it is important to identify women at risk for psychological distress (Brandão et al., 2017). Among coping strategies used by these patients, there have been reports of the importance of accepting the diagnosis and engaging in physical activities providing social and emotional support (Lashbrook et al., 2018).

Coping research has recently suggested the concept of “coping flexibility” to overcome the lack of diversity and fluidity of coping, as considered in more classical research (Kato, 2012). This concept is based on the transactional theory presuming that coping may change over time according to the demands of a specific stressful situation (Lazarus and Folkman, 1984). As such, coping flexibility corresponds to the ability to discontinue an ineffective coping approach and produce and implement an alternative one (Kato, 2012). Accordingly, in the context of trauma, resilience would be fostered by the ability to flexibly engage in different coping strategies as needed, and not by a single type of coping (Bonanno, 2004, 2005; Bonanno et al., 2011).

According to a review on coping flexibility (Cheng et al., 2014), the most widely used instruments for measuring this construct are the self-rated Flexible Goal Adjustment Scale (FGAS; Brandtstädter and Renner, 1990) and the Coping Flexibility Questionnaire (CFQ; Cheng, 2001). The FGAS is a 15-item questionnaire, rated in a 5-point Likert scale, to assess the ability to modify coping goals according to changing environments. It assesses coping according to two perspectives: *assimilation*, i.e., seeking to change one’s development conditions according to personal preferences (*Tenacious Goal Pursuit* subscale); and *accommodative flexibility*, i.e., indicating the adjustment of individual preferences to situational limits (*Flexible Goal Adjustment* subscale). Yet, studies using this scale have noted that it fails to adequately distinguish between its two subscales (Henselmans et al., 2011). The CFQ is an open-ended, situation-based measure. It assesses, through a 6-point Likert scale, coping responses to a series of stressful life events. Flexible coping is obtained as an individual coping profile, indicating the frequency of using different strategies according to each stressful situation. Limitations of this scale have been shown in cross-cultural studies, with differences in the use of coping strategies according to culture (Basińska et al., 2021). Other scales have also been proposed to assess flexible coping. The Coping Flexibility Scale (CFS; Kato, 2012) is based on the dual-process model of coping flexibility previously proposed in the FGAS. The flexible goal adjustment process was refined by suggesting an additional process that precedes it. According to Kato (2012) one should be able to recognize that a strategy no longer works before implementing an alternative (i.e., adaptive coping process). The CFS assesses two flexible coping processes across the *Evaluation coping* and *Adaptive coping* subscales. Similar to the limitations of CFQ, the CFS has shown to be susceptible to cultural influences (Basińska, 2015). The Coping Flexibility Questionnaire (COFLEX; Vriezekolk et al., 2012) is a 13-item instrument that includes two dimensions of coping flexibility: *versatility*, the capability of using the different available coping resources according to the circumstances, and *reflective coping*, the capability of generating and considering coping options, and estimating the suitability of a coping strategy in a given situation. While there was preliminary evidence of the validity of the versatility dimension, for reflective coping it could not be firmly established. Moreover, Basińska et al. (2021) raised another weakness of the scale, based on the fact that its development and the selection of items was guided by theory and selected by researchers, instead of being produced by patients.

In the specific context of potentially traumatic events, Bonanno et al. (2011) examined the notion of flexible coping according to concepts of perceived ability. They hypothesized that effectively coping with trauma involves the flexible use of two coping processes: *forward focus*, the perceived ability to move beyond the trauma, and *trauma focus*, the perceived ability to process the trauma. These two coping strategies are assessed by the Perceived Ability to Cope with Trauma scale (PACT; Bonanno et al., 2011). This is a self-rated questionnaire, explicitly designed to assess the perceived ability to cope with potentially traumatic events, providing a measure of coping flexibility. The PACT displayed adequate reliability and validity across Israeli and American samples (Bonanno et al., 2011), demonstrating cross-cultural adequacy. It has been mainly used in studies of coping in highly trauma-exposed samples (Bonanno et al., 2011; Park et al., 2015; Bartholomew et al., 2017; Pinciotti et al., 2017; Sullivan and Wade, 2019), in potentially traumatic life events such as adjustment to college (Bonanno et al., 2011; Galatzer-Levy et al., 2012; Saita et al., 2017), grief severity in older widows and widowers (Knowles and O’Connor, 2015), experience with the COVID-19 pandemic (Zhou et al., 2020; Brivio et al., 2021), and in breast cancer patients (Hamama-Raz et al., 2012; Pat-Horenczyk et al., 2016; Brivio et al., 2021). The present study aimed to adapt the European Portuguese version of the PACT scale and validate it for patients with breast cancer.

## Materials and Methods

### Participants

This study was conducted at the Champalimaud Clinical Centre under the multicenter clinical study---BOUNCE (Predicting Effective Adaptation to Breast Cancer to Help Women to BOUNCE Back).^2^ This study included the completion of questionnaires assessing quality of life, mood, and personal characteristics. Patients with a recent diagnosis of stage I-III histologically confirmed breast cancer, and eligible for systemic treatment, were recruited at their first clinical visit to the oncologist and invited to participate in the study before starting any systemic treatment. Eligibility criteria included: women 18–70 years of age at the time of diagnosis, histologically confirmed invasive breast cancer, tumor stages I—III, local treatment with surgery with or without adjuvant radiation therapy, any type of systemic treatment. Exclusion criteria were: distant metastasis; history of another malignancy or contralateral invasive breast cancer within the last 5 years, with the exception of cured basal cell carcinoma of skin or carcinoma *in situ* of uterine cervix; history of early onset (i.e., < 40 years of age) mental disorder (i.e., schizophrenia, psychosis, bipolar disorder, major depression) or severe neurologic disorder (i.e., neurodegenerative disorder); other serious concomitant diseases such as clinically significant (i.e., active) cardiac disease (e.g., congestive heart failure, symptomatic coronary artery disease or uncontrolled cardiac arrhythmia) or myocardial infarction within the last 12 months; major surgery for a severe disease or trauma which could affect patient’s psychosocial wellbeing (e.g., major heart or abdominal surgery) within 4 weeks prior to study entry, or lack of complete recovery from surgery.

### Measures

#### Sociodemographic and Lifestyle Questionnaire and Medical Data

This questionnaire was developed specifically for this study to assess sociodemographic and lifestyle variables, such as age, educational level, marital status, and employment status, as reported by the participants.

#### Perceived Ability to Cope With Trauma

The PACT Scale (Bonanno et al., 2011) is a 20-item self-report measure of beliefs about the capability to manage traumatic sequelae. Answers are given in a Likert-type scale that ranges from 1 (“Not at all able”) to 7 (“Extremely able”). The original version indicated the presence of two subscales: Forward focus and Trauma focus. Forward focus (12 items) was identified by the authors as the perceived ability to move beyond the trauma, i.e., assessing coping abilities related to keeping plans and goals, attending to the needs of others, thinking optimistically, remaining calm, reducing painful emotions, and being able to laugh. The Trauma Focus subscale (8 items) was proposed to measure the perceived ability to process the trauma, through a full experience of the emotional and cognitive significance of a stressful and potentially traumatic event. An algorithm index of flexibility is calculated to estimate the ability to engage in both types of coping (Bonanno et al., 2011). The coping flexibility score is computed by (1) adding the average scores of the trauma focus and the forward focus subscales to create a total coping score, (2) creating a polarity score by taking the absolute values of the difference between the standardized trauma focus and forward focus subscale scores, and (3) subtracting the polarity score from the total coping score to generate a coping flexibility score. Greater scores mean greater coping flexibility strategies. In the current study, analyses will cover the two PACT scales (Trauma Focus and Forward Focus), as well as the total coping and the flexibility scores. In the original version, the Cronbach alpha was 0.91 for the Forward Focus and 0.79 for the Trauma Focus (Bonanno et al., 2011).

#### Cancer Behavior Inventory-Brief Version

The Cancer Behavior Inventory-Brief Version [CBI-B; (Heitzmann et al., 2011); Portuguese Version by Pereira et al. (2021)] is a brief version derived from the Cancer Behavior Inventory-Long (33 items). The instrument consists of 12 items and is a measurement of self-efficacy for behaviors related to coping with cancer. Following each item is a Likert-type scale that ranges from 1 (“not at all confident”) to 9 (“totally confident”), reflecting the degree of confidence that cancer related coping behaviors will be performed. The total score of the scale is obtained by summing the scores of all items, where higher scores refer to higher self-efficacy in coping with cancer. In the Portuguese validation study, the Cronbach alpha was 0.88 (Pereira et al., 2021) while in the present study it was 0.86, confirming high internal consistency.

#### European Organization for Research and Treatment of Cancer Quality of Life Questionnaire-Core 30

The European Organization for Research and Treatment of Cancer Quality of Life Questionnaire Core-30 [EORTC QLQ-C30; (Aaronson et al., 1993); Portuguese Version by Pais-Ribeiro et al. (2008)] is 30-item questionnaire to assess health-related quality of life (QoL) in patients with cancer, from the moment of diagnosis to long-term survivorship. The questionnaire combines five functional subscales (physical, role, emotional, cognitive, and social), three symptom subscales (fatigue, nausea and vomiting, and pain), a global health/QoL subscale, and a few single items assessing other symptoms frequently reported by cancer patients (dyspnea, insomnia, appetite loss, constipation, and diarrhea) as well as the perceived financial impact of the disease. All items are scored in 4-point Likert-type scales ranging from 1—“not at all” to 4—“very much,” except two items of the global health/QoL subscale, that use a modified 7-point linear analog scale (from 1—“poor” to 7—“excellent”). Each multi-item scale includes a different set of items, i.e., no item appears in more than one scale. Higher scores received from the global health/QoL subscale indicate higher quality of life, whereas higher scores obtained from the functional or the symptom scales/items indicate lower quality of life. For this study, namely for assessing convergent validity, we only used the global quality of life score. In the Portuguese validation study, the Cronbach’s alpha for the global quality of life was 0.88 (Pais-Ribeiro et al., 2008), and 0.87 in the present study.

#### Hospital Anxiety and Depression Scale

The Hospital Anxiety and Depression Scale [HADS; (Zigmond and Snaith, 1983); Portuguese Version by Pais-Ribeiro et al. (2007)] is a 14-item self-report measure designed to assess severity of depression and anxiety symptoms, in two separate subscales. Items are answered in a 4-point Likert-type scale response category (ranging between 0 and 3). Higher scores in the total scale indicate greater psychological distress. In the original Portuguese validation study (Pais-Ribeiro et al., 2007), including patients with cancer, Cronbach’s alpha was 0.76 for the anxiety subscale and 0.81 for the depression subscale. In this study, only the total scale was calculated with a Cronbach’s alpha of 0.89.

### Procedures

**FIGURE 1:**
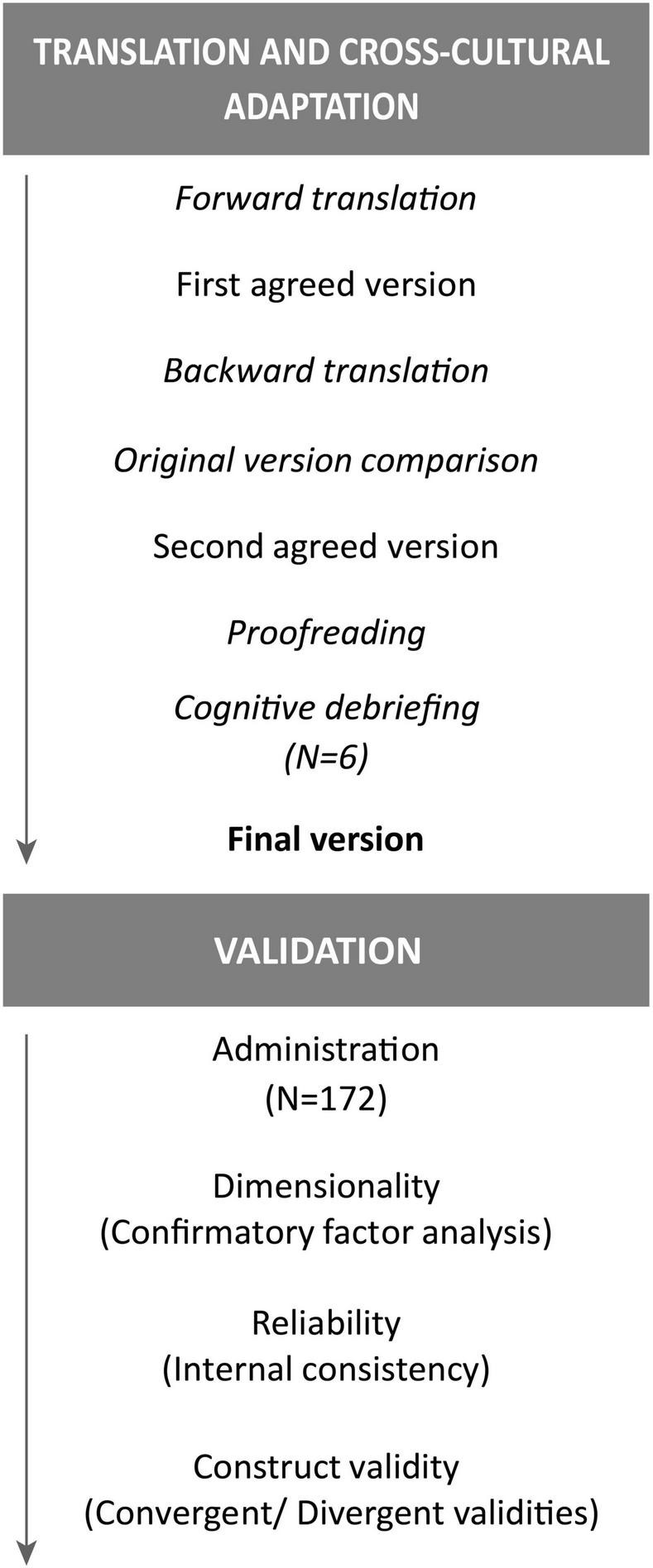
Flowchart of the cross-cultural adaptation and validation of the Perceived Ability to Cope with Trauma (PACT).

Permissions to translate the PACT scale were obtained from the original authors by the BOUNCE study project manager. We then followed the International Test Commission Guidelines for Translating and Adapting Tests (Gregoire, 2018). Briefly, forward translation of the original scales from English to European Portuguese was performed separately by two bilingual experts in Psychology of Portuguese dominant language, resulting in two forward translated versions of the scale. A translation panel composed of mental health and oncology specialists who had not been involved in any of the translations then conducted a reconciliation of the two forward translations. The reconciled translation was then backward translated into English by two bilingual translators, of English dominant language, that worked independently and were not involved in the original translations. This was followed by comparison of the backward translated versions by the translation panel, thus creating a consensus backward translation version, that was compared against the source language by the initial translation team, to confirm similarity between the two versions and address any potential major differences in the consensus backward translation by adjustments of the consensus forward translation. The resulting harmonized version of the consensus forward translation was tested among representatives of the target population and language group (6 Portuguese patients with breast cancer), in a cognitive debriefing session to determine if the respondents understood the questions being asked and if there were words or phrases that were not familiar. No significant difficulties were reported in the debriefing session. The input from these patients considered only the replacement of some words for synonyms with a higher frequency in European Portuguese, so that it could be easily understood. For example, “*evento*” (Portuguese word for event) was replaced by “*acontecimento*” (Portuguese word for happening), as event may indicate a special moment like a party or wedding. This happened both in the test instructions and in some items. After the input from these patients, the translation was reviewed, and proofreading was conducted to ensure that minor errors were corrected, resulting in the final Portuguese translation of the scale (for an overview see Figure 1).

Study procedures and protocol were reviewed and approved by the Ethics Committee of our institution. Informed consent was obtained from all participants, and the study was conducted in accordance with the Declaration of Helsinki. Data was collected, stored, and processed in accordance with ethical principles and applicable international, EU and National legislation, in particular the General Data Protection Regulation (EU GDPR).

### Data Analysis

Descriptive statistics for sociodemographic and psychometric data included means and standard deviations (SD), minimum and maximum absolute values, percentages, skewness and kurtosis, that obtained using the Statistical Package for the Social Sciences (SPSS, Version 27.0; IBM SPSS, Inc., Chicago, IL). To assess dimensionality, confirmatory factor analysis (CFA) was calculated using JASP version 0.14.1,^3^ which is built on the R-package lavaan.^4^ A model was specified according to the original two-factor structure (Bonanno et al., 2011). Diagonally Weighted Least Squares estimation method was employed because of the ordinal scale structure and because of the relatively small sample size, according to current recommendations (Jöreskog and Sörbom, 1989; Li, 2016; Gana and Broc, 2018). Model fit indices included: (a) non-significant χ^2^; (b) the comparative fit index (CFI) and the Tucker–Lewis index (TLI), with values ≥0.90 and ≥0.95 indicating good and very good model fit, respectively; and (c) the Root Mean Square Error of Approximation (RMSEA), and the Standardized Root Mean Square Residual (SRMR) indexes, with values ≤0.08 and ≤0.05 indicating acceptable and very good model fit, respectively (Hair, 2011; Gana and Broc, 2018). Item local adjustment was analyzed through the inspection of factor loadings (λ), that represent the strength of the relationship among the latent variable and the observed variable. Significant (*p* ≤ 0.05) factor loadings with λ ≥ 0.40 are considered as a good indicator of the quality of the items (Gana and Broc, 2018). Reliability was examined using the McDonald’s omega and the Cronbach’s alpha, with coefficients ≥0.70 suggesting good factor reliability (Hair, 2011), and using the corrected item-total correlation, with values above 0.30 suggesting good inter-item correlation (Nunnally and Bernstein, 1994). To assess construct validity, we used Pearson’s correlation coefficients between the PACT scores and scores on measures of self-efficacy (CBI-B) and QoL (QLQ-C30) for convergent validity, and psychological distress (HADS) for divergent validity. Independent samples *t*-tests were used to compare means between groups. Results with alpha-level (*p*) < 0.05 were considered statistically significant.

## Results

### Descriptive Statistics

Our sample included 172 women with early or locally advanced, non-metastatic breast cancer, treated with either chemotherapy (*n* = 99) or endocrine therapy (*n* = 73), for whom sociodemographic data and clinical characteristics are presented in Table 1. The overall mean age (±SD) was 50.7 (±9.1), but the endocrine therapy group (53.4 ± 9.3) was significantly older than the chemotherapy group [48.7 ± 8.4; *t*_(170)_ = –3.47, *p* = 0.001], as expected. Most patients had completed higher education (47.1% with a bachelor’s degree and 26.7% with a graduate degree); 74.4% were married; and 83.1% were employed.

**TABLE 1:** Sociodemographic and Clinical characteristics of the sample.

Demographic and clinical characteristics (*n* = 172)	*n*	%
Age, mean (SD)	50.66 (9.08) [Min. (22); Max. (70)]	
**Age group**		
≤40 y	23	13.4
41–50 y	73	42.4
51–60 y	47	27.3
>60 y	29	16.9
**Highest level of education**		
Primary	5	2.9
Lower secondary	9	5.2
Higher secondary	31	18.0
Post-secondary non-graduate	81	47.1
Graduate degree	46	26.7
**Marital status**		
Single/Engaged	20	11.6
Married	128	74.4
Divorced/widowed	24	14.0
**Employment status**		
Employed	143	83.1
Unemployed/housewife	11	6.4
Retired	18	10.5
**Treatment**		
Chemotherapy (CT)	99	57.6
Endocrine Therapy (ET)	73	42.4

Descriptive statistics (means, standard deviations, kurtosis, skewness) of individual PACT items are shown in Table 2. The percentage of endorsement for each item is also provided, showing an overall tendency for higher value ratings (7—“extremely able”).

**TABLE 2:** Individual PACT item summaries for the total sample.

Item	Statistics			Percentage of endorsement							
	M (SD)	Sk	Ku	1	2	3	4	5	6	7	Total
1	5.24 (1.51)	–0.97	0.64	3.5	2.9	4.7	15.9	21.2	30.6	21.2	100
2	5.58 (1.43)	–1.34	1.61	2.3	2.9	3.5	9.9	15.2	38.6	27.5	100
3	5.39 (1.39)	–0.85	0.43	1.2	3.5	3.5	17.0	20.5	31.0	23.4	100
4	5.23 (1.46)	–0.92	0.48	2.4	3.5	6.5	14.1	21.8	33.5	18.2	100
5	5.48 (1.29)	–1.08	1.29	1.2	2.3	5.3	7.6	27.5	34.5	21.6	100
6	5.09 (1.37)	–0.39	–0.42	0.6	3.0	8.9	21.3	24.3	24.9	17.2	100
7	5.62 (1.55)	–0.94	–0.03	1.2	2.9	8.2	11.2	15.3	19.4	41.8	100
8	5.55 (1.49)	–1.12	0.95	2.3	3.5	2.3	12.9	19.9	25.7	33.3	100
9	5.42 (1.18)	–0.85	0.99	0.6	1.8	3.5	12.9	28.2	35.9	17.1	100
10	5.54 (1.35)	–0.79	0.07	0.6	1.8	5.9	14.2	19.5	28.4	29.6	100
11	5.18 (1.46)	–0.72	–0.11	1.2	4.8	7.7	16.1	19.6	32.1	18.5	100
12	5.94 (1.11)	–1.11	1.04	0.0	1.2	1.8	8.8	15.9	35.3	37.1	100
13	5.36 (1.30)	–0.72	0.05	0.0	4.1	4.1	15.9	22.9	32.9	20.0	100
14	5.70 (1.21)	–0.84	0.16	0.0	1.2	4.8	10.1	20.8	32.7	30.4	100
15	5.44 (1.33)	–0.86	0.37	0.6	2.9	5.3	13.5	21.2	34.1	22.4	100
16	5.74 (1.24)	–1.03	0.86	0.6	0.6	5.3	8.8	19.4	32.9	32.4	100
17	5.85 (1.15)	–0.81	0.03	0.0	0.6	3.0	10.1	20.7	29.0	36.7	100
18	5.36 (1.19)	–0.67	0.49	0.6	1.2	4.1	16.6	27.2	33.7	16.6	100
19	5.47 (1.38)	–0.73	–0.05	0.6	2.4	5.9	15.4	20.1	27.2	28.4	100
20	5.00 (1.36)	–0.53	–0.00	1.2	4.1	7.1	21.2	27.6	25.3	13.5	100

*For each item of the PACT scale, the mean, standard deviation, and the percentage of endorsement for each possible item score (range 1–7) is displayed. PACT, Perceived Ability to Cope with Trauma; M, Mean; SD, Standard deviation; Sk, Skewness; Ku, Kurtosis.*

### Dimensionality

**FIGURE 2:**
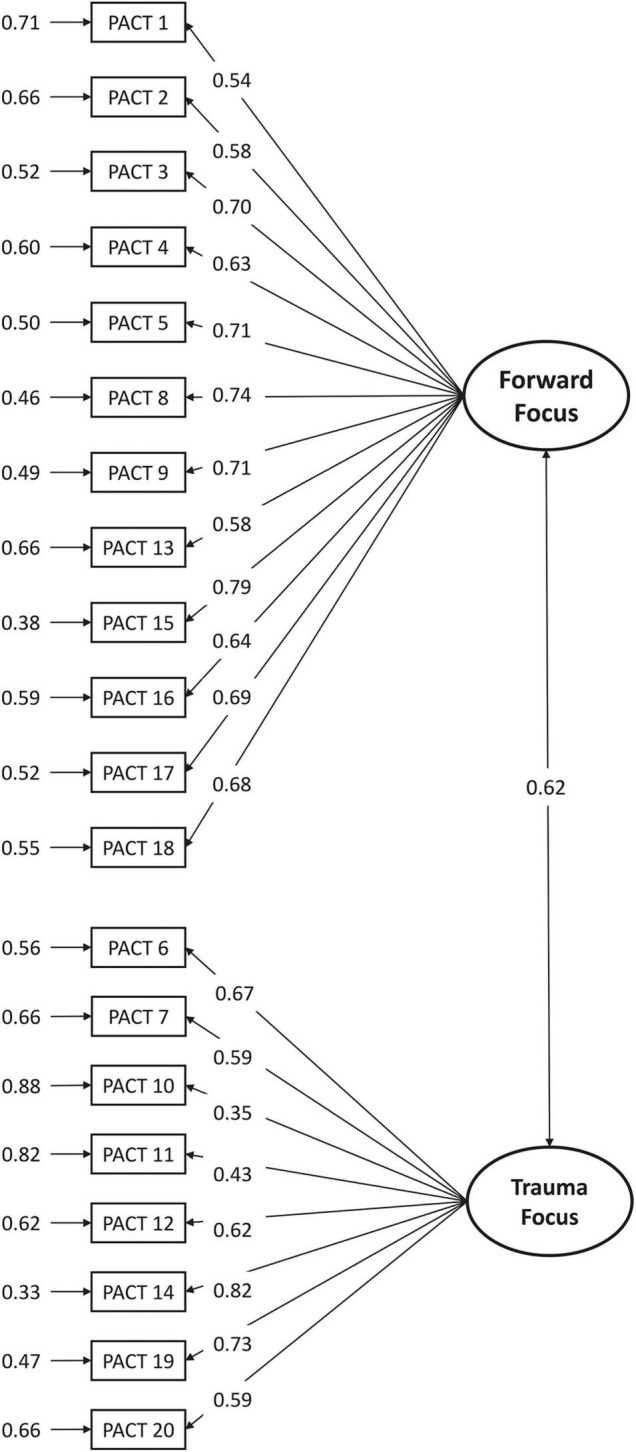
Confirmatory factor analysis of the two-factor model of the PACT. Standardized coefficients and measurement errors are shown.

A CFA of the PACT two-factor model suggested for the original version was conducted. The general model indicated a good model fit through adequate goodness-of-fit indices: χ^2^(169) = 166.3, *p* = 0.54; CFI = 1.00; TLI = 1.00; and RMSEA = 0.00, 90% CI: 0.00–0.03; SRMR = 0.08. Overall, all items presented good local adjustment, with loadings ranging from λ = 0.43 (item 11) to λ = 0.82 (item 14), except for item 10 that presented a loading of 0.35 (Figure 2). We decided to retain the item as our model had showed an overall very good fit of the model, that would not improve with elimination of item 10.

### Reliability

Internal consistency was estimated using using the McDonald’s omega and the Cronbach’s alpha. Factor 1 (“Forward focus” scale) showed an excellent reliability (ω = 0.91, 90% CI: 0.89–0.92; α = 0.90, 90% CI: 0.88–0.92). The values remained stable with removal of any item (ω: 0.89–0.91; α: 0.89–0.90), and corrected item-total correlations ranged between 0.51 and 0.75. Lower, but good, values were found for Factor 2 (“Trauma focus” scale): ω = 0.82, 90% CI: 0.79–0.86; α = 0.83, 90% CI: 0.79–0.86. Again, the values remained stable with removal of any item (0.79–0.82), and corrected item-total correlations ranged between 0.48 and 0.68.

### Construct Validity

Table 3 shows correlations between the four PACT scores and other self-report measures selected to test convergent and divergent validity. When compared with the CBI-B scale, the PACT scores had adequate convergent validity (Factor 1: *r* = 0.35, *p* < 0.001; Factor 2: *r* = 0.32, *p* < 0.001; total coping: *r* = 0.41, *p* < 0.001; flexibility: *r* = 0.26, *p* = 0.01). The QoL scale (QLQ-C30) was also adequately correlated with Factor 1 (*r* = 0.34, *p* < 0.001), total coping (*r* = 0.27, *p* = 0.001), and flexibility (*r* = 0.17, *p* = 0.03), but and was not significantly correlated with Factor 2 (*r* = 0.15, *p* = 0.07). Regarding divergent validity, some PACT factors correlated negatively with psychological distress as measured by HADS (Factor 1: *r* = –0.38, *p* < 0.001; total coping: *r* = –0.25, *p* = 0.002; flexibility: *r* = –0.19, *p* = 0.01) whereas PACT Factor 2 was not significantly correlated with distress (*r* = –0.09, *p* = 0.28).

**TABLE 3:** Convergent and divergent validities.

	PACT Factor 1 *(forward focus)*	PACT Factor 2 *(trauma focus)*	PACT total coping	PACT flexibility
		
		Convergent validity		
CBI-B—total score	0.36***	0.32***	0.41***	0.26**
EORTC QLQ-C30 QoL	0.34***	0.15*^NS^*	0.27**	0.17*

		**Divergent validity**		

HADS—total score	–0.38***	–0.09*^NS^*	–0.25**	–0.20*

PACT, Perceived Ability to Cope with Trauma; CBI-B, Cancer Behavior Inventory brief version; HADS, Hospital Anxiety and Depression Scale; EORTC QLQ-C30, European Organization for Research and Treatment of Cancer Core Quality of Life Questionnaire. ***p < 0.001; **p < 0.01; *p < 0.05; NS, non-significant.

### Trauma Focus, Forward Focus, Total Coping, and Coping Flexibility Among Women With Breast Cancer

Overall, PACT scores in our sample were *M* = 65.49; *SD* = 11.17 for Factor 1—*Forward focus*; *M* = 43.58; *SD* = 7.18 for Factor 2—Trauma Focus; *M* = 10.94; *SD* = 1.57 for Total coping and *M* = 9.85; *SD* = 2.76 for Flexibility. No differences were found when comparing the two clinical samples (CT and ET) on the PACT scale subscores (Table 4), revealing similar coping strategies and flexibility irrespective of the breast cancer treatment plan.

**TABLE 4:** Performance on the PACT scale.

	Chemotherapy group	Endocrine therapy group	*t*	*p*
PACT Factor 1—forward focus, mean (SD)	64.64 (11.79)	66.64 (10.25)	–1.14	0.26
PACT Factor 2—trauma focus, mean (SD)	43.14 (7.45)	44.16 (6.81)	–0.89	0.37
PACT total coping, mean (SD)	10.80 (1.61)	11.11 (1.51)	–1.22	0.22
PACT flexibility, mean (SD)	9.56 (2.95)	10.25 (2.44)	–1.61	0.11

*Comparisons between groups were carried out by independent sample t-tests.*

## Discussion

The present study describes the cross-cultural translation and adaptation of the PACT scale into European Portuguese, aiming at evaluating its psychometric properties, in terms of dimensionality, reliability (internal consistency), and construct (convergent and divergent) validity, in a sample of patients with breast cancer. Our results supported a two-factor structure for the PACT scale, consistent with the forward focus and trauma focus subscales proposed by the original authors (Bonanno et al., 2011). Noteworthy, the factorial structure of the original PACT scale was demonstrated in assessments of a potentially highly trauma-exposed Israeli sample and a group of American college students, in Hebrew and English, respectively (Bonanno et al., 2011). The other known published factorial structure of the PACT refers to its Italian version (Saita et al., 2017). The purpose of Saita et al. (2017) was to examine the factorial structure of the PACT scale in an Italian sample that shared characteristics with the individuals involved in the original validation study, thus including college students not directly exposed to potentially traumatic events but that could potentially present high levels of distress. The final most appropriate factor structure of the Italian version resulted in a total of 14 items, instead of the original 20 (Saita et al., 2017). The authors explain this difference based on different cultural aspects, highlighting the challenges of cross-cultural measurements (Saita et al., 2017).

Our study investigated the psychometric properties of the PACT scale in a different and novel sample: women recently diagnosed with breast cancer, that also constitutes a potentially traumatic event. Even though Portuguese shares a common Latin origin with Italian, representing two similar cultures, we were able to confirm the two-factor solution and preserve the 20-item scale used originally by Bonanno et al. (2011). In our results, item 10 (“Reduce my normal social obligations”) presented a borderline factor loading, but we decided to retain it as our model showed an overall very good fit. In the Italian version, item 10 was eliminated due to very low factor loading in an Exploratory Factor Analysis (Saita et al., 2017). We believe that the concept of “social obligations” may have different meanings between Hebrew/English and Portuguese/Italian: while in the first it would be related to social responsibilities, in the latter it could be interpreted as social events, leading participants to rate how they would be able to reduce their usual social life events following a potentially traumatic event. Methodologically, as was also pointed out by Saita et al. (2017), these studies highlight the challenges associated with adapting existing instruments to a different culture following the etic approach (Tran et al., 2018). The etic approach argues that psychological processes are universal in nature and that instruments developed in a specific population could be applied to another (Saita et al., 2017; Tran et al., 2018). In the process of cross-cultural adaptation of the PACT scale, we assumed *a priori* that coping flexibility would be similar across the original Hebrew/English and Portuguese. Overall, our results confirmed both conceptual and statistical equivalences between the two versions.

Dimensionality findings were supported by the reliability results. The Portuguese version of the PACT was reliable when used to examine breast cancer patients. Factor 1 (“Forward focus” scale—12 items) showed an excellent reliability (ω = 0.91; α = 0.90), whereas a good value (ω = 0.82; α = 0.83) was found for Factor 2 (“Trauma focus” scale—8 items). The values remained stable with removal of any item on both factors, and the item-total correlations presented high levels, therefore confirming the theoretical structure of the two subscales. The two factors were moderately correlated. Similar results were displayed in the original version, where the 12 item-Forward focus also presented excellent reliability (0.91) and the 8 item-Trauma focus had a lower value (0.79) (Bonanno et al., 2011). Likewise, the Italian version of the PACT revealed a higher reliability of factor 1 (0.87) than of factor 2 (0.70) (Saita et al., 2017). Another study involving patients with breast cancer found similar results for forward focus (0.93) and trauma focus (0.74) (Hamama-Raz et al., 2012).

Concerning convergent and divergent validity, all PACT scores were related to high self-efficacy to coping with cancer (CBI-B), and most were related to high perceived QoL (QLQ-C30) and to low psychological distress (HADS). Our findings are consistent with previous research that demonstrated convergent validity with measures of positive cognitive–emotional regulation, ego resiliency, and optimism (Bonanno et al., 2011), thus suggesting that individuals with greater perceived coping ability and flexibility are more likely to experience positive emotions. On the other hand, as stated by the original authors, PACT scores are expected to show mild inverse associations with negative affect and with anxious or avoidant attachment (Bonanno et al., 2011), therefore confirming divergent validity. Our results confirmed that the PACT scores are positively associated with better adjustment to the diagnosis of cancer, and inversely related to psychological distress.

Furthermore, the two clinical samples (CT and ET) had similar results on all the PACT scale subscores, proving similar coping strategies and coping flexibility irrespective of the breast cancer treatment plan. The PACT and other psychosocial measures assessment were performed just after the diagnosis, and therefore either before the start of chemotherapy or within 2 weeks from the start of the endocrine therapy. Although patients were already aware of their therapeutic plan, no interpretations about coping strategies to deal with the trauma of the specific treatments and related side-effects can be made. The diagnosis of breast cancer is known to be particularly stressful, leading women to adapt coping strategies to address this challenge (Matthews et al., 2017).

Even though cancer is not an acute traumatic event, multiple studies have sought to measure the presence of PTSD in patients with cancer (Cordova et al., 2001, 2007, 2017; Mehnert and Koch, 2007; Foster et al., 2009; Arnaboldi et al., 2017; Matthews et al., 2017). It is important to highlight that, in the context of cancer, the threat is not only related to the present but also to the future, and patients are thus required to manage worry or distress about future health (Pat-Horenczyk et al., 2016). Experiencing positive emotions and reducing painful emotions, maintaining plans and goals, thinking optimistically, remaining calm, and being able to laugh, as well as processing the traumatic event, allows individuals to evoke powerful changes in their emotional trajectory (Bonanno, 2005, 2004; Bonanno et al., 2011). In this regard, the PACT scale has been shown to be useful in measuring coping flexibility and in moderating the impact of heightened trauma exposure to breast cancer (Hamama-Raz et al., 2012; Pat-Horenczyk et al., 2016; Brivio et al., 2021).

Hamama-Raz et al. (2012) aimed to explore the causes affecting the decision of patients with breast cancer to participate in group intervention, after the end of adjuvant therapy, based on an approach to enhance resilience. Significantly higher levels of coping flexibility on PACT were reported by women who did not show an interest in the group intervention, relative to those who participated (Hamama-Raz et al., 2012). The authors suggest that women employing appropriate and psychologically healthy ways of coping are more likely to reveal resilience and emotional regulation, and may therefore perceive group therapy as not necessary or redundant (Hamama-Raz et al., 2012). In another post-cancer treatment study, Pat-Horenczyk et al. (2016) employed the PACT scale to assess the level of coping flexibility of female breast cancer patients over a 2-year period. The objectives of their study were to identify different post-treatment adaptation profiles, factors that predicted the adaptation profiles, and trajectories and transitions in the adaptation profiles. Four post-cancer treatment adaptation profiles were suggested by the authors: distressed, resistant, constructive growth, and struggling growth (Pat-Horenczyk et al., 2016). In the specific case of coping flexibility, it significantly predicted the likelihood of belonging to a particular profile, i.e., higher levels of flexibility increased the odds of being classified as either struggling or constructive growths, as well as decreased the likelihood of belonging to the distressed profile (Pat-Horenczyk et al., 2016).

In a very recent study, Brivio et al. (2021) showed that coping flexibility contributed significantly to manage the positive and negative affect in patients with cancer during the COVID19 pandemic in Italy. The positive states were evidenced by the PACT Trauma focus subscale, confirming that the perceived ability to focus on processing the trauma is associated with positive states (Brivio et al., 2021). Altogether, these results proved that higher levels of coping flexibility enhances resilience and emotional regulation (Hamama-Raz et al., 2012), are associated both post-traumatic growth and distress (Pat-Horenczyk et al., 2016), and helps activate a more positive outlook and think realistically about COVID-19 (Brivio et al., 2021), among breast cancer patients. Noteworthy, the available PACT versions are limited to English, Hebrew, and Italian, with studies performed in patients with breast cancer performed with the latter two. Our methodological approach, including analysis of the PACT scale validity; reliability; and responsiveness, correspond to the recommended quality criteria for measurement properties of health status questionnaires (Terwee et al., 2007) and of assessment tools in cancer patients (Tian et al., 2019).

In the present study we assessed women with early breast cancer just before starting treatment, aiming at evaluating the potentially traumatic experience of receiving a breast cancer diagnosis. Our sample was subdivided according to treatment plan (chemo vs. endocrine therapy). Some limitations should consequently be noted, mainly related to psychometric assessments that could not be performed. First, the sample size represents a limitation that prevented the performance of a differential item functioning analysis or a multigroup CFA. These analyses would enable us to show the extent to which an item might be measuring different abilities between members of different groups or to psychometrically determine whether the PACT would elicit a similar response pattern across our two subsamples. Secondly, test-retest reliability could not be performed as a retest assessment would probably lead to a different condition while answering to the PACT questions, as patients would be at a treatment phase and coping would most likely be related to treatment and not to diagnosis. We therefore consider that assessing test-retest reliability could lead to misinterpretations and opted not to include it. Future studies should include patients with other types of cancer or different cancer stages, or samples related to other traumatic events, thus providing additional information about the PACT and its applicability for Portuguese patients.

Despite these limitations, the current study contributes to the literature about cross-cultural adaptation and measurement by examining, for the first time, the psychometric properties of the PACT scale in a Portuguese sample of women with breast cancer. Our results indicate that the original two-factor structure is applicable in the current sample. Moreover, a similar organization of coping styles is maintained, and a latent measure of coping flexibility can be calculated. Using this measure in clinical practice may contribute to understand how patients cope with potentially traumatic events, thus helping to provide proper interventions to achieve better psychological adjustments.

## Data Availability Statement

The raw data supporting the conclusions of this article will be made available by the authors, without undue reservation.

## Ethics Statement

The studies involving human participants were reviewed and approved by the Ethics Committee of the Champalimaud Foundation. The patients/participants provided their written informed consent to participate in this study.

## Author Contributions

RL and AO-M conceived and designed the work. RL and DF were responsible for the translation process of the PACT scale. DF and BS acquired the data, including assessment of eligibility criteria of patients participating in the study. RL, BC, SA, DF, and AO-M analyzed and interpreted data. BC and DF extracted clinical and demographic data with input from RL, BS, and AO-M. RL, BC, and AO-M drafted the manuscript, which was critically revised by the remaining authors for important intellectual content. AO-M supervised the research and acts as corresponding author. All authors contributed to the article and approved the submitted version.

## Conflict of Interest

AO-M was national coordinator for Portugal of a non-interventional study (EDMS-ERI-143085581, 4.0) to characterize a Treatment-Resistant Depression Cohort in Europe, sponsored by Janssen-Cilag, Ltd. (2019–2020), is recipient of a grant from Schuhfried GmbH for norming and validation of cognitive tests, and is national coordinator for Portugal of trials of psilocybin therapy for treatment-resistant depression, sponsored by Compass Pathways, Ltd. (EudraCT number 2017-003288-36 and 2020-001348-25), and of esketamine for treatment-resistant depression, sponsored by Janssen-Cilag, Ltd. (EudraCT NUMBER: 2019-002992-33). The remaining authors declare that the research was conducted in the absence of any commercial or financial relationships that could be construed as a potential conflict of interest.

## Publisher’s Note

All claims expressed in this article are solely those of the authors and do not necessarily represent those of their affiliated organizations, or those of the publisher, the editors and the reviewers. Any product that may be evaluated in this article, or claim that may be made by its manufacturer, is not guaranteed or endorsed by the publisher.

## Footnotes

1
https://gco.iarc.fr

2
https://www.bounce-project.eu/

3
https://jasp-stats.org

4
http://lavaan.ugent.be
